# Identifying the cause of thermal droop in GaInN-based LEDs by carrier- and thermo-dynamics analysis

**DOI:** 10.1038/s41598-020-74585-w

**Published:** 2020-10-15

**Authors:** Dong-Pyo Han, Gyeong Won Lee, Sangjin Min, Dong-Soo Shin, Jong-In Shim, Motoaki Iwaya, Tetsuya Takeuchi, Satoshi Kamiyama, Isamu Akasaki

**Affiliations:** 1 Faculty of Science and Technology, Meijo University, Nagoya, 468-8502 Japan; 2 Department of Photonics and Nanoelectronics, Hanyang University ERICA, Ansan, 15588 Gyeonggi-do Korea; 3 Akasaki Research Center, Nagoya University, Nagoya, 464-8603 Japan

**Keywords:** Lasers, LEDs and light sources, Inorganic LEDs

## Abstract

This study aims to elucidate the carrier dynamics behind thermal droop in GaInN-based blue light-emitting diodes (LEDs) by separating multiple physical factors. To this end, first, we study the differential carrier lifetimes (DCLs) by measuring the impedance of a sample LED under given driving-current conditions over a very wide operating temperature range of 300 K–500 K. The measured DCLs are decoupled into radiative carrier lifetime (*τ*_*R*_) and nonradiative carrier lifetime (*τ*_*NR*_), via utilization of the experimental DCL data, and then very carefully investigated as a function of driving current over a wide range of operating temperatures. Next, to understand the measurement results of temperature-dependent *τ*_*R*_ and *τ*_*NR*_ characteristics, thermodynamic analysis is conducted, which enables to look deeply into the temperature-dependent behavior of the carriers. On the basis of the results, we reveal that thermal droop is originated by the complex dynamics of multiple closely interrelated physical factors instead of a single physical factor. In particular, we discuss the inherent cause of accelerated thermal droop with elevated temperature.

## Introduction

Following the successful growth of GaN crystals and p-type GaN with the help of a low-temperature (LT) buffer layer and low-energy electron beam irradiation by H. Amano et al.^1,2^ the remarkable development of AlGaInN-based light-emitting diodes (LEDs) employing multiple-quantum-well (MQW) active regions in recent decades has made them commercially available for various applications, such as headlamps of vehicles, visible light communications, bio-sterilizers, and micro-LED displays^3–5^. Particularly, GaInN-based blue LEDs covered with yellow phosphors are being used widely as general white-light sources^6,7^. Despite the recent significant progress, however, AlGaInN-based LEDs still suffer from several efficiency degradations, such as the external quantum efficiency (EQE, *η*_EQE_) gradually degrading with increasing emission wavelength, driving current, and operating temperature, often referred to as “green-gap,” “efficiency droop,” and “thermal droop,” respectively^8–10^. Specifically, the thermal droop in GaInN-based blue LEDs has recently become a major research topic because of the continuous demand on white-light emitters that work efficiently in any operating environment^10–14^. It is noted that the above three efficiency issues are mainly caused by degradation of the internal quantum efficiency (IQE, *η*_IQE_), as they are not deeply related to degradation of the light-extraction efficiency (LEE, *η*_LEE_)^15^. Recall *η*_EQE_ = *η*_LEE_ × *η*_IQE_^3,14^. The thermal droop in GaInN-based blue LEDs can also be explained by the gradual reduction of the IQE with elevating operating temperature. Therefore, a careful analysis of the IQE is essential in investigating the thermal droop.

To date, many carrier recombination and transport mechanisms have been proposed as the potential cause of the thermal droop phenomena in GaInN-based blue LEDs, including the Shockley–Read–Hall (SRH) recombination, hole localization, thermionic emission, local Joule heating, Auger recombination, and tunneling leakage^13–19^. Still, the mechanism that causes such thermal droop, which may consist of several physical factors, is not fully understood and remains debatable.

The IQE of an LED can be expressed in terms of carrier lifetimes as follows^9,20^:

1 $$\eta_{{{\text{IQE}}}} = \frac{{\tau_{{\text{R}}}^{ - 1} }}{{\tau_{{\text{R}}}^{ - 1} + \tau_{{{\text{NR}}}}^{ - 1} }},$$

where *τ*_R_ and *τ*_NR_ represent the radiative and nonradiative carrier lifetimes, respectively. Concerning Eq. (1), theories explaining the mechanism of thermal droop should include one of the following three with temperature: (i) increase of *τ*_R_ (i.e., decrease of the radiative recombination rate), (ii) decrease of *τ*_NR_ (i.e., increase of the nonradiative recombination rate), or (iii) increase of *τ*_R_ and decrease of *τ*_NR_ occurring simultaneously. Hence, it is highly required to separately measure *τ*_R_ and *τ*_NR_, i.e., decoupling the carrier lifetimes, at various operating temperatures and driving currents, to elucidate the mechanism that causes the thermal droop.

The IQE degradation has been explained in several studies by carrier recombination and transport dynamics in LEDs via measurements of the differential carrier lifetimes (DCLs, *τ*_D_). Several techniques have been conducted to measure DCLs thus far, of which the most frequently adopted is the optically pumped small-signal method because it is simple to set up experimentally and conduct measurements with no further sample fabrication^21–23^. However, there are times when an optically pumped operating condition does not fully demonstrate the carrier dynamics under typical electrically driven operating conditions, which gives an incomplete outlook of the physics behind the IQE degradation^24^. The S-parameters measurement technique, which uses a network analyzer, has been proposed to overcome this issue with an advantage of a fully electrically driven condition^24,25^. However, in contrast to the optical-pump approach, this method requires a complex experimental setup and further sample fabrication. Alternatively, a long historical method of impedance measurement with electrically injected RF small signals can provide a simple experimental setup, an easy sample preparation, and fully electrically driven operating conditions, enabling the DCL to be easily and exactly obtained^26^. Aside from the measurement method issue, equivalent circuits and carrier rate equations are required to decouple *τ*_R_ and *τ*_NR_ from the DCL data^21–25^. Because the IQE is determined from several complex carrier dynamics in and out of MQWs [recall *η*_IQE_ = *η*_RE_ × *η*_IE_, where *η*_RE_ is the radiative efficiency (RE) and *η*_IE_ is the injection efficiency (IE)]^3,14^, it is crucial to adopt the exact equivalent circuit and carrier rate equation that consider all the factors mentioned above comprehensively.

From the perspective of investigating the temperature-dependent properties of LEDs, the analysis of thermodynamic properties is very useful for understanding the thermal droop. Under electrical operation, the energy supplied to an LED is consumed in several ways, mainly by light or heat generation. The thermal energy generated by nonradiative recombination can have a fatal effect on the LED performance. Specifically, further energy transfer to carriers in the MQWs introduces “hot carriers,” resulting in an acceleration of thermionic emission and space-charge-limited current^3^. That is, the IQE and the voltage efficiency (VE, *η*_VE_) are significantly affected by the heat generation inside the LED. Since the thermal droop is apparently associated with the thermal energy of carriers in the MQWs, the thermodynamic analysis can provide informative data.

Recently, we have successfully demonstrated the technique to measure the DCLs using an impedance analyzer and a method to decouple *τ*_R_ and *τ*_NR_ with an equivalent circuit and carrier rate equation. We have carefully analyzed the measured values of *τ*_R_ and *τ*_NR_ with combining the energy loss analysis, i.e., thermodynamic analysis, as a function of driving current at room temperature, which consequently provides a comprehensive outlook on the carrier dynamics responsible for the efficiency droop in GaInN-based LEDs^27,28^. More importantly, this technique only requires a simple experimental setup and sample preparation, which allows both the measurement of the DCL and the analysis of thermodynamics over a wide range of operating temperatures. Thus, we believe that widening the range of operating temperatures in our previous study can provide insights into the carrier dynamics responsible for the thermal droop in GaInN-based LEDs.

This study aims to elucidate the carrier dynamics responsible for the thermal droop in GaInN-based blue LEDs, which may consist of several mechanisms, by decoupling the multiple physical factors. To this end, first, we use an impedance analyzer to obtain the impedance of a sample over a very wide range of operating temperatures (300–500 K), depending on the driving current. The DCLs are extracted with an equivalent circuit from frequency-dependent impedance data. Carrier lifetimes are decoupled into *τ*_R_ and *τ*_NR_ by using the experimental IQE and DCL data and then very carefully investigated as a function of driving current over a wide operating-temperature range. Next, to deepen our understanding of the measurement results of temperature-dependent *τ*_R_ and *τ*_NR_ characteristics, thermodynamic analysis is conducted, which enables to look deeply into the temperature-dependent behavior of the carriers. Collectively, on the basis of DCL and thermodynamic analysis, we try to comprehensively elucidate the mechanism behind thermal droop in GaInN-based LEDs in aspect of both the carrier dynamics and thermodynamics. We expect that the analysis performed in this study can provide a different perspective on the mechanism behind thermal droop in GaInN-based LEDs.

## Experimental results, analysis, and discussion

### Thermal droop behavior of GaInN-based LEDs

**Figure 1:**
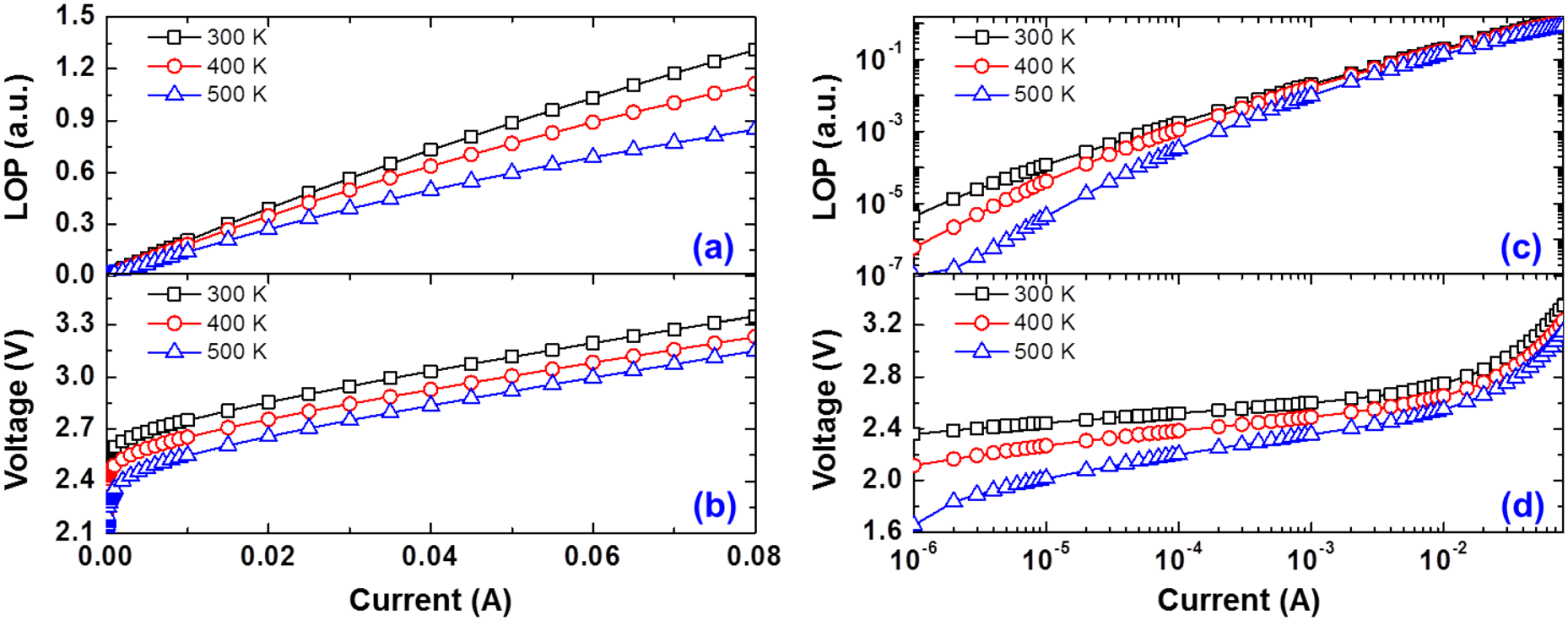
The light output powers (LOPs) and applied voltages (*V*) of the sample under investigation as functions of driving current at various operating temperatures: (**a**, **b**) on linear scales; (**c**, **d**) on logarithmic scales.

To observe the thermal droop behavior of the sample under investigation, we measured its light output powers (LOPs) and applied voltages (*V*) as functions of driving current over the operating temperature range, 300–500 K. Figure 1a,b show the corresponding linear plots. Apparently, both LOPs and *V* decrease with an elevation in temperature throughout the entire current range, exhibiting the typical temperature-dependent electrical and optical characteristics of GaInN-based LEDs. Specifically, the LOP at 500 K driven at a usual operating current (50 mA) is only 68% of that at 300 K, suggesting a thermally degrading device performance. The decrease in applied voltage with elevated temperature in the entire current region, particularly the change in slope in the logarithmic curve at a low current regime (10^–6^ A–10^–4^ A) (Fig. 1d), suggests not only the change in bandgap energy but also the change in predominant carrier conduction and recombination mechanisms since it represents an ideality factor of LED device^3,29^, which is discussed in detail later in this section.

**Figure 2:**
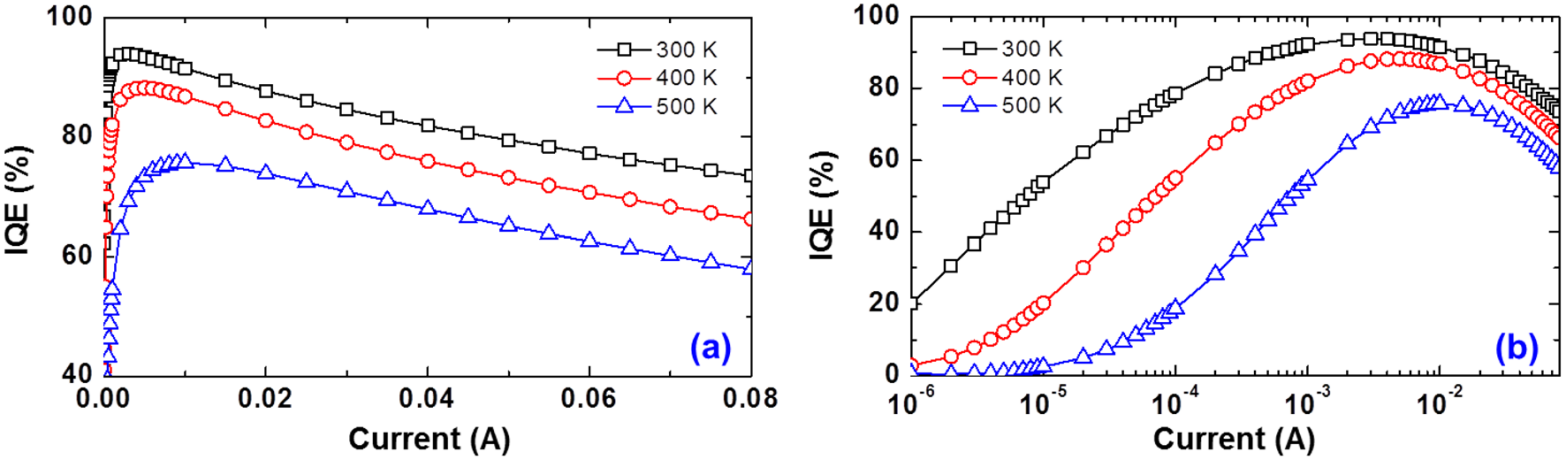
The internal quantum efficiencies (IQEs) of sample under investigation as functions of driving current depending on the chamber temperature, plotted on (**a**) linear and (**b**) logarithmic scales.

To investigate the thermal droop in detail, we measured the IQEs as a function of driving current at various operating temperatures. Figure 2a,b show their linear and semi-log plots, respectively. Note that the IQEs in Fig. 2 were evaluated by the method presented in our previous paper^3,30^. As expected, the IQE gradually decreases with elevating chamber temperature over the entire current range. Particularly, the IQE of the sample at 500 K driven at 50 mA was only 72% of that at 300 K, suggesting that the IQE degradation is the main cause of the thermal droop, i.e., the LOP degradation in Fig. 1a. It is noteworthy that the driving current at the maximum IQE increases with an increasing operating temperature, whereas the efficiency-droop behavior, i.e., negative slopes of the IQE at the high-current regime (Fig. 2a), show similar values for all temperatures.

### DCLs measurement and separation into ***τ***_***R***_ and ***τ***_***NR***_

**Figure 3:**
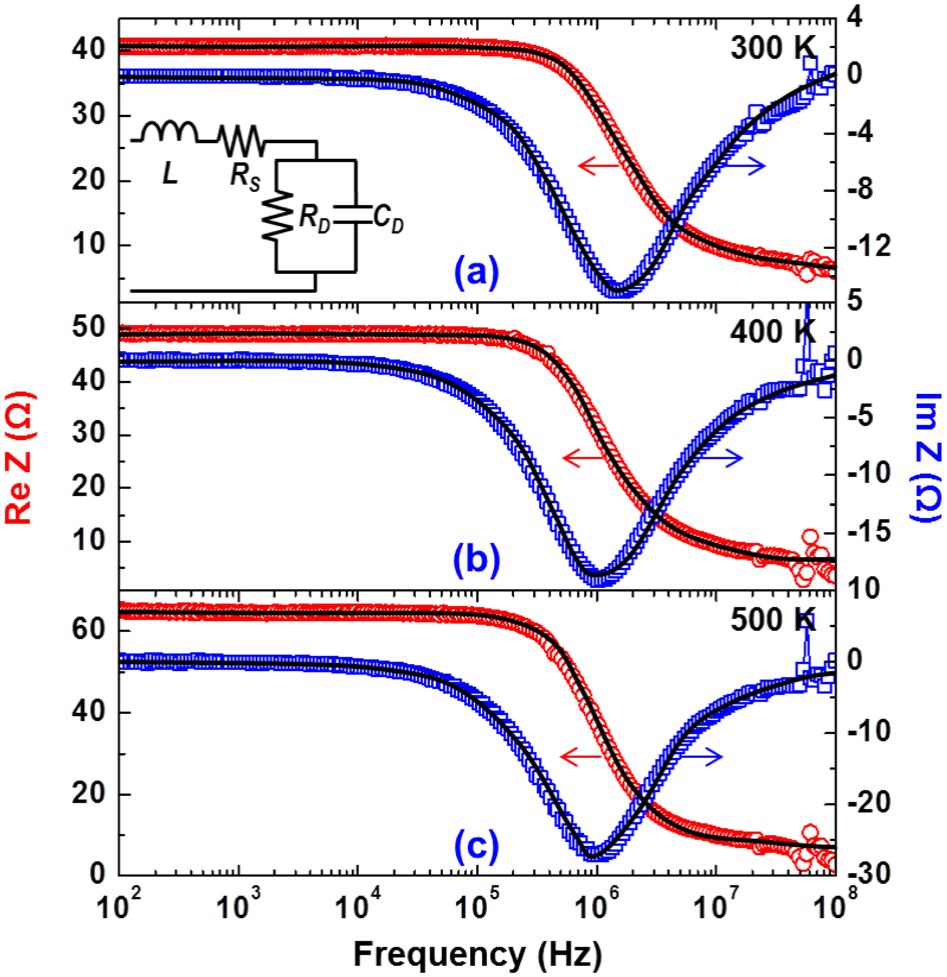
Real and imaginary parts of the frequency-dependent impedance (Re Z and Im Z) at a driving current of 1 mA under operating temperatures of (**a**) 300 K, (**b**) 400 K, and (**c**) 500 K. The inset shows the small-signal equivalent circuit of the LED device. The black solid lines are the fitting curves using the equivalent circuit.

Next, to obtain DCLs of the sample, we measured the real and imaginary parts of the frequency-dependent impedance (Re Z and Im Z) as a function of driving current at 300, 400, and 500 K. Figure 3a–c are given to show Re Z and Im Z values at a driving current of 1 mA under operating temperatures of 300, 400, and 500 K, respectively. As mentioned above, the impedance measurement, purely electrical, was used in this study to obtain the DCLs. Thus, we could use the electrical equivalent circuit (inset of Fig. 3a) corresponding to the small-signal response of the LED, which consists of a junction resistor (*R*_D_), a junction capacitor (*C*_D_), a series resistor (*R*_*S*_), and a parasitic inductor (*L*). The DCL is defined as the derivative of the total recombination rate in the LED (*R*) with respect to carrier density in the active region (*n*) at a certain carrier density (*n*_0_):

2 $$\tau_{{\text{D}}}^{ - 1} = \left. {\frac{dR}{{dn}}} \right|_{{n_{0} }}$$

which can be represented as a circuit element with *τ*_*D*_ = *R*_*D*_*C*_*D*_^31–33^. Briefly, the DCL can be obtained by extracting *R*_D_ and *C*_D_ of the equivalent circuit, which is possible through fitting of Re Z and Im Z vs. frequency curves with the equivalent circuit. In Fig. 3a–c, we can see that the measured impedance data are fitted very well by the equivalent circuit, as represented by the black solid lines. The fitting parameters used in Fig. 3a–c are summarized in Table 1.

**Table 1:** Summary of fitting parameters used in Fig. 3a–c.

Temperature	*L* (nH)	*R*_*S*_ (Ω)	*R*_*D*_ (Ω)	*C*_*D*_ (pF)
300 K (Fig. 3a)	7.53	5.94	35.41	78.91
400 K (Fig. 3b)	7.53	3.53	46.07	74.24
500 K (Fig. 3c)	7.53	2.81	63.11	67.85

**Figure 4:**
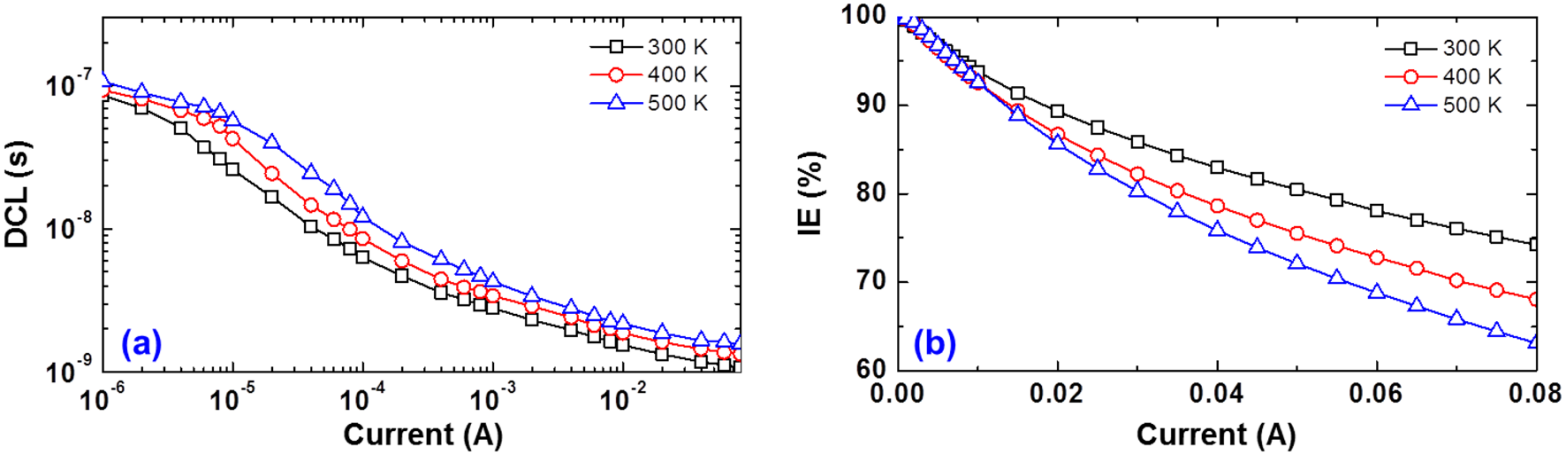
(**a**) Differential carrier lifetimes (DCLs) and (**b**) injection efficiencies (IEs) of sample under investigation as a function of driving current at various operating temperatures.

We conducted the same fitting for every impedance data to investigate the driving-current- and operating-temperature-dependent DCLs. Figure 4a shows the logarithmic plots of the DCLs as a function of driving current at various operating temperatures on a logarithmic scale. There is an obvious decrease in the DCLs as the driving current is increased for all temperatures, whereas they increase slightly with the elevated operating temperature at the same current injection, similar to previous results^16,24^. By integrating the measured DCL over the current, *n* at a driving current can be estimated:

3 $$n\left( I \right) = \frac{{\eta_{{{\text{IE}}}} \left( I \right)}}{{qV_{{{\text{eff}}}} }}\int_{0}^{I} {\tau_{{\text{D}}} \left( {I^{\prime}} \right) \, } dI^{\prime}$$

where *q* is the elementary charge and *V*_eff_ is the effective volume contributing to carrier recombination that can be different from the physical volume of the MQWs. Note that the recent studies have revealed that only 3%–15% of the MQW physical volume is used for the radiative recombination because nonideal physical factors, such as carrier localization, hole transport, and the quantum-confined Stark effect (QCSE), contribute to the reduction of *V*_eff_^3,34^. For the sample here, we assume that 10% physical volume of the MQWs is used for *V*_eff_. In Eq. (3), information on the IE is important to obtain *n* as it represents the carrier density in *V*_eff_, whereas the DCL contains information of recombinations occurring both inside and outside the active region.

Typically, recombination in LEDs is described by the *ABC* model. This simplistic model only takes into account the SRH, radiative, and Auger recombination inside MQWs, i.e., recombinations occurring inside the active region. Meanwhile, recent studies revealed that recombinations that occurs outside the active region predominates at high-current injection in GaInN-based LEDs. In this study, we thus utilized *AB* + *f*(*n*) model^30,35,36^, which is frequently adopted to comprehensively explain recombinations occurring both inside and outside the active region. In this model, the IQE is written in terms of the carrier rate equation as following,

4 $$\eta_{{{\text{IQE}}}} = \frac{{B\left( {n,T} \right)n^{2} }}{{A\left( {n,T} \right)n + B\left( {n,T} \right)n^{2} + f\left( {n,T} \right)}},$$

where *T* is the temperature, *A*(*n,T*) and *B*(*n,T*) are the SRH nonradiative recombination and the bimolecular radiative recombination coefficient in MQWs. Note that the recombination coefficients are expressed as a function of *n* and *T,* and the general carrier loss term, *f*(*n*,*T*) is employed to express the carrier dynamics at high current injection including the Auger recombination, carrier overflow, thermionic emission, and spill-over, that is typically composed of terms higher than third order, i.e., *f*(*n*,*T*) = *C*(*n*,*T*)*n*^3^ + *D*(*n*,*T*)*n*^4^ +…. On basis of Eq. (4), the IE is defined as [*A*(*n,T*)*n* + *B*(*n,T*)*n*^2^]/[ *A*(*n,T*)*n* + *B*(*n,T*)*n*^2^ + *f*(*n,T*)]^35^ and we calculated the IE using a method used in Refs.^28^ and^36^. Particularly, the *C*(*n*,*T*)*n*^3^ is included in the IE because it consisted of not only the pure Auger recombination^37^, but also the phase-space filling (PSF) effect on the Auger recombination^30^, the phonon-assisted recombination^38^, the indirect Auger recombination^39^, the hot carrier generation^40^, and carrier emissions to the vacuum level^41^, which results in carrier recombination outside the active region. Figure 4b shows the linear plot of the IEs as a function of driving current at various operating temperatures. The IE shows similar behavior within a few mA after the onset of IQE droop, regardless operating temperature (*I* <  ~ 10 mA). Meanwhile, the decrease in IE with elevated temperature in the high current region (~ 10 mA < *I*) is observed, which seems to be one of the possible origins of thermal droop. Since *f*(*n*,*T*) is the sum of terms higher than third order, the higher-order terms are expected to be predominate as injected current increases. Thus, we infer that the terms higher than fourth order, representing the carrier escaping from the MQWs, have a greater impact on the IE at high-current injection. More details on this are discussed later in next section.

**Figure 5:**
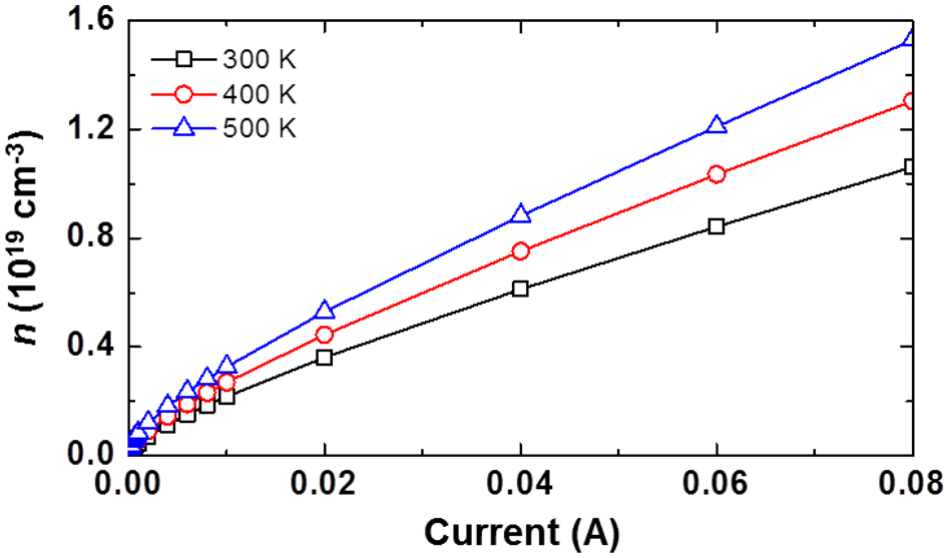
Carrier density in the active region (*n*) as a function of driving current at various temperatures.

Meanwhile, Fig. 5 shows a linear plot of *n* as a function of driving current at various operating temperatures. There is an obvious increase in *n* as the driving current and the operating temperature are increased.

To clarify the carrier dynamics responsible for the thermal droop, we decouple the DCLs into *τ*_R_ and *τ*_NR_. Accordingly, *τ*_R_ and *τ*_NR_ can be expressed in terms of the IQE, the IE, the driving current, and the DCL as follows:

5 $$\tau_{{\text{R}}} = \frac{{\eta_{{{\text{IE}}}} }}{{\eta_{{{\text{IQE}}}} I}}\int_{0}^{I} {\tau_{{\text{D}}} \left( {I^{\prime}} \right) \, } dI^{\prime}$$

6 $$\tau_{{{\text{NR}}}} = \frac{{\eta_{{{\text{IQE}}}} }}{{1 - \eta_{{{\text{IQE}}}} }}\tau_{{\text{R}}}$$

**Figure 6:**
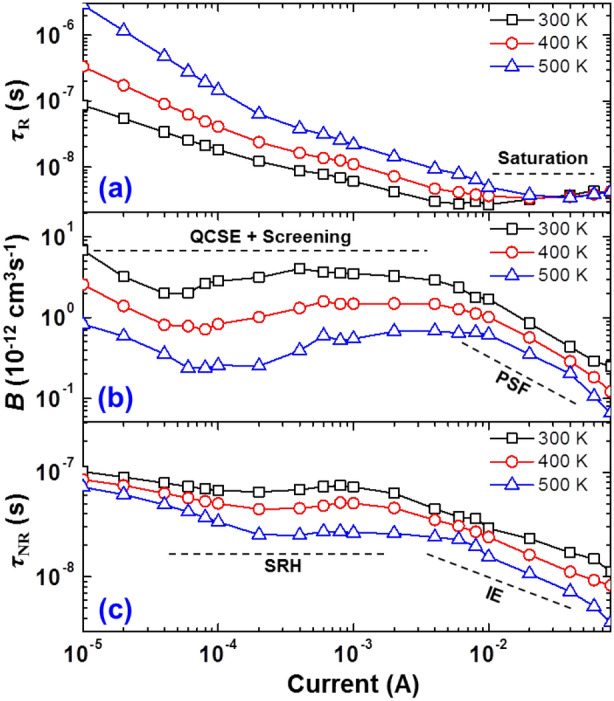
(**a**) Radiative carrier lifetime (*τ*_R_), (**b**) bimolecular recombination coefficient (*B*), and (**c**) nonradiative carrier lifetime (*τ*_NR_) as a function of driving current at various temperatures.

Equations (5) and (6) give the values for *τ*_R_ and *τ*_NR_ using the experimental data of IQEs in Fig. 2, DCLs in Fig. 4a, and IEs in Fig. 4b. Figure 6a and c show the log–log plots of *τ*_R_ and *τ*_NR_ as a function of driving current at various temperatures.

In Fig. 6a, it is seen that *τ*_R_ decreases gradually with increasing driving current and decreasing operating temperature in the low-current regime. In contrast, *τ*_R_ becomes saturated to a similar level in the high-current regime regardless of the operating temperature. Since the *τ*_R_ is composed of *n* and recombination probability [Recall *τ*_R_ = (*Bn*)^−1^], the temperature-dependent characteristics of *B* can exhibit an inherent property of radiative recombination. Figure 6b shows *B* as a function of driving current depending on operating temperatures, which is obtained by combining the data in Fig. 5 and Fig. 6a. The driving-current-dependent characteristics of *B* seems to be typical: the QCSE associated with the screening effect is predominant in the low-current regime (below the current at the IQE peak) and the PSF is predominant in the high-current regime (above the current at the IQE peak)^29,31^. Notably, *B* gradually decreases with elevated temperature over the entire current range, implying a gradual reduction of the radiative recombination probability as the operating temperature is increased.

It is seen in Fig. 6c that *τ*_NR_ shows a dissimilar tendency to *τ*_R_ as the operating temperature and the driving current are increased. It yields a nearly constant value depending on driving current in the low-current regime, whereas there is a sharp decrease in the high-current regime. The different driving-current-dependent characteristics of *τ*_NR_ obviously results from the fact that the predominant carrier dynamics is changed, i.e., from the SRH in the low-current regime to the IE in the high-current regime. Collectively, on the basis of Fig. 6a–c, we can infer that the thermal droop is originated by the complex dynamics of multiple closely interrelated physical factors, i.e., the *τ*_R_, *τ*_NR_, IE and PSF, instead of a single physical factor.

**Figure 7:**
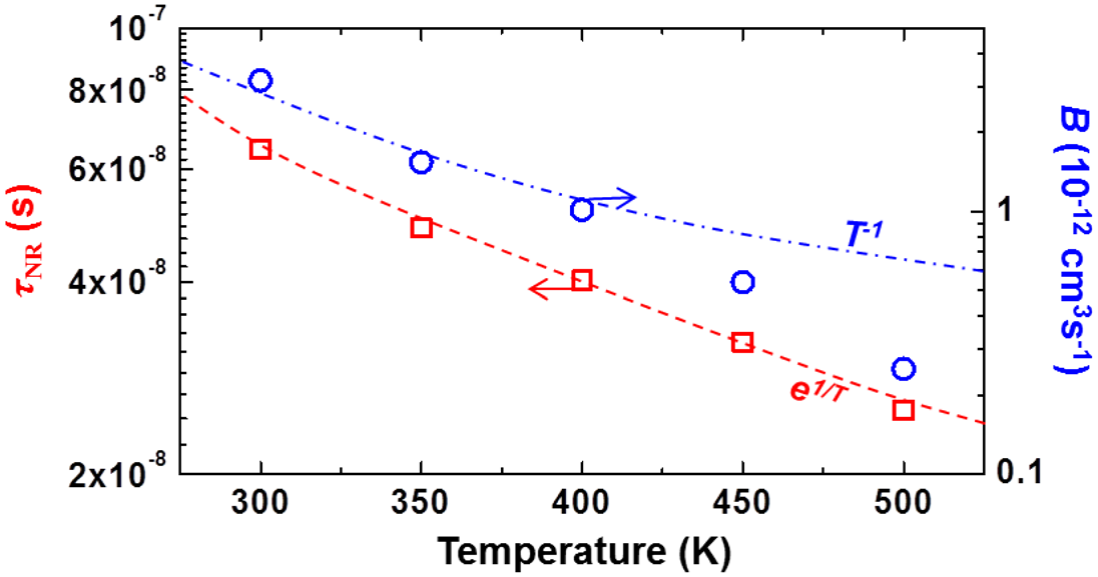
Nonradiative carrier lifetime (*τ*_NR_) and bimolecular recombination coefficient (*B*) at 200 μA as a function of temperature. The dashed lines are theoretical predictions.

The temperature-dependent SRH recombination lifetime (*τ*_SRH_) is theoretically expressed in terms of temperature as follows^21,38^:

7 $$\tau_{{{\text{SRH}}}} = \tau_{0} \left[ {1 + \cosh \left( {\frac{{E_{{\text{T}}} - E_{{{\text{Fi}}}} }}{{k_{{\text{B}}} T}}} \right)} \right]$$

where *τ*_0_ is a constant related to the trap density, *E*_T_ is the energy of the traps, *E*_Fi_ is the intrinsic Fermi level and *k*_B_ is the Boltzmann constant. From Eq. (7), the temperature-dependent behavior of *τ*_SRH_ can be approximately written by *τ*_SRH_ ∝ e^1/^^*T*^. In contrast, from ref. 20, the temperature-dependent behavior of *B* for 2-D quantum well can be approximately expressed as *B* ∝ *T*^−1^. In Fig. 7, *τ*_NR_ and *B* at 200 μA are plotted as a function of operating temperature and compared with the theoretical predictions represented by dashed lines. Note that the values of *τ*_NR_ and *B* at low current injection are compared because *τ*_NR_ is considered as *τ*_SRH_ and the PSF effect on *B* is negligible under this situation. As shown in Fig. 7, the experimental results of the temperature-dependent behavior of *τ*_NR_ at 200 μA, i.e., *τ*_SRH_, are well matched to the theoretical prediction over an entire operating temperature range. On the other hand, the experimental results of the temperature-dependent behavior of *B* at 200 μA seem to be matched to the theoretical prediction only at the temperature range of 300–400 K. That is, the discrepancy to the dashed line is exhibited at the operating temperature above 400 K, which gradually increases with the elevated temperature. It suggests that *B* is reduced more than the theoretical prediction as the temperature is elevated further.

Recent studies have shown that the sharp reduction in *B* with increasing driving current, i.e., the PSF effect, triggers the reduction of the IE. Specifically, the PSF induces the carrier injection rate to exceed the total recombination rate in the MQWs, which accelerates the carrier escaping the MQWs, leading to the decrease in IE^3,9,28^ and the efficiency droop. Hence, *τ*_R_ and *τ*_NR_ are not independent of each other, but are closely related. In this point of view, the further reduction of *B* more than the theoretical prediction can promote the thermal droop. Besides, the mechanism behind the thermal droop seems to be intricately combined with *τ*_R_, *τ*_NR_, the PSF, and the IE on the basis of the data presented so far. Therefore, we believe that there are factors simultaneously governing *τ*_R_, *τ*_NR_, the PSF, and the IE, which may present the key towards identifying the mechanism behind the thermal droop.

### Thermodynamic analysis of LEDs

**Figure 8:**
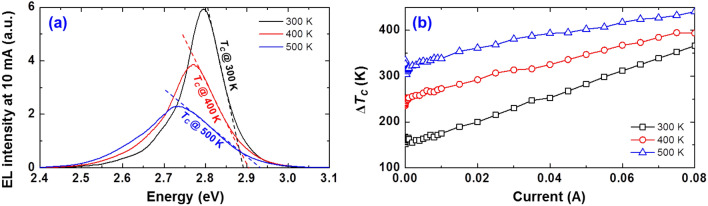
(**a**) Electroluminescence (EL) spectrum at 10 mA and (**b**) *∆T*_*C*_ as a function of driving current at various temperatures.

To identify the key towards unraveling the mechanism behind the thermal droop, we conduct analysis of the thermodynamic properties of LEDs since it is very useful for investigating their temperature-dependent behaviors of carrier. First, we consider looking into the LED deeper by measuring the electroluminescence (EL) spectrum as a function of driving current at various temperatures since the EL spectrum typically corresponds to the energy distribution of carriers in the MQWs. As an example, Fig. 8a shows the EL spectrum at 10 mA under various temperatures. Here, the carrier temperature (*T*_*C*_) can be obtained from the high-energy slope of the EL spectrum as shown in Fig. 8a^42^. Fig. 8b depicts a plot of *∆T*_*C*_ as a function of driving current at various temperatures. *∆T*_*C*_ represents the additional carrier energy gained from an extrinsic source. The values are defined as follows: *∆T*_*C*_ at 300 K = *T*_*C*_ − 300 K, *∆T*_*C*_ at 400 K = *T*_*C*_ − 400 K, and *∆T*_*C*_ at 500 K = *T*_*C*_ − 500 K. It is noteworthy that the negative slope at extremely low-current regime (*I* < 0.1 mA) in Fig. 8b is considered to be due to the screening of the QCSE associated with the screening effect and/or the carrier localization-delocalization. At the current injection above 0.1 mA, the injected carriers are expected to hold the Boltzmann distribution of MQWs because the recombination rate at the localized states is rapidly saturated in GaInN-based blue LEDs^43,44^. As shown in Fig. 8b, *∆T*_*C*_ increases gradually with temperature and driving current, implying that the additional energy of the carriers in the MQWs increases gradually with both. From the data, we believe that the carriers are distributed in abnormally high-energy states under high current and high temperature. Theoretically, the carrier distribution in the abnormally high-energy states accelerates the mismatch between electron and hole in *E-k* space^20^, resulting in the further reduction of radiative recombination probability^3,9^. Moreover, such carrier distribution in energy space can make an impact on the IE characteristics because the probability of carriers escaping the MQWs increases very rapidly when the carriers receive sufficient energy to overcome the band offset. In short, the carriers abnormally energized by an extrinsic source can induce a noticeable reduction of the radiative recombination and the IE, thereby accelerating the thermal droop. Therefore, it is important to identify an extrinsic energy source that provides additional thermal energy to the carrier.

**Figure 9:**
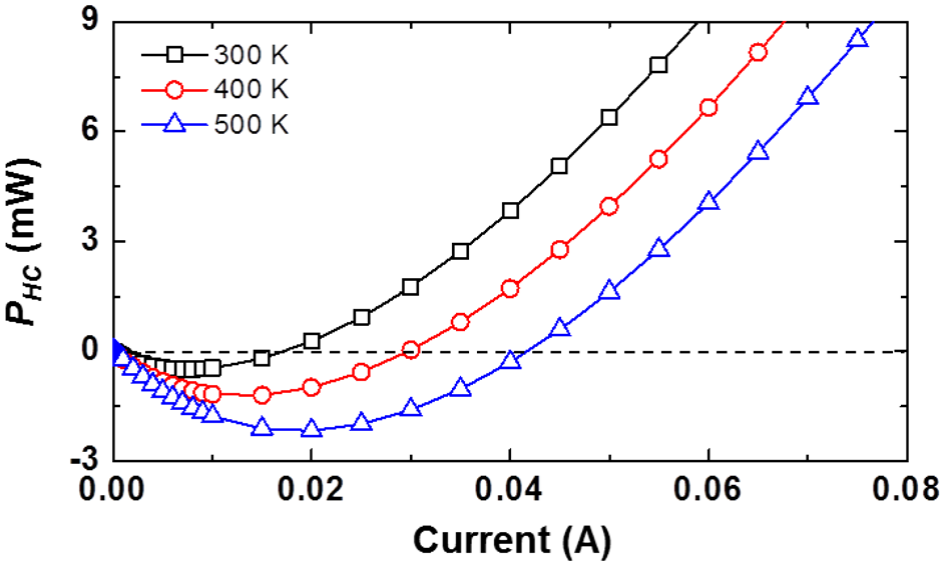
*P*_*HC*_ of sample under investigation as a function of driving current at various temperatures.

Recently, it was experimentally verified how the carriers in the MQWs are energized via evaluations of the heating or cooling power (*P*_HC_) of carriers with lattices, typically known as the electroluminescent cooling or Peltier heating in heterostructure semiconductors^13,45,46^. By definition, *P*_HC_ can be approximately expressed as follows:

8 $$P_{{{\text{HC}}}} = \left( {V - IR_{S} - \frac{{h\nu_{{{\text{EL}}}} }}{q}} \right) \times I \times \eta_{{{\text{IQE}}}} ,$$

where *R*_*S*_ is the series resistance and *hv*_EL_ is the average photon energy of the EL. Using the measurement data in Figs. 1, 2, and 7, we obtain *P*_HC_ of the sample, whose plot as a function of driving current under various temperatures is shown in Fig. 9. Apparently, the value of *P*_HC_ becomes negative as soon as the carriers gain energy from the lattice^3,28,45^. *P*_HC_ becomes more negative at higher temperatures, which implies that the carriers gain a large amount of energy from the lattice as the temperature increased. Here, the source of *P*_HC_ would most likely to be the SRH recombination because it increases with temperature^46^. Moreover, the pronounced lattice vibration at high temperature facilitates the efficient energy transfer from the lattice to the carriers, namely, the energy transfer efficiency from lattice to the carrier increases. As the current increases further, the slope of *P*_HC_ turns from negative to positive after the peak negative magnitude of *P*_*HC*_ for all case of operating temperature, implying that the dominant transport mechanism has transitioned from the diffusion injection to the carrier escaping from the MQWs since carriers gain enough energy to overcome the band offset between the well and the barrier from the SRH recombination^3,28,45,46^. On the basis of this analysis, we can obtain an important information that the IE and *B* properties are significantly influenced by the carrier distribution in the energy space, by mean of that the energy generated mainly by the SRH recombination is transferred to the carrier in the MQWs through lattice vibration, which is enhanced with elevated temperature. Consequently, the increase in the SRH recombination with increasing temperature not only decreases the *τ*_NR_, but also has a great impact on the *τ*_R_ and the IE, resulting in the acceleration of the thermal droop. The main mechanisms that cause the thermal droop as mentioned above, i.e., *τ*_R_, *τ*_NR_, PSF, and the IE, are physically related closely to each other.

**Figure 10:**
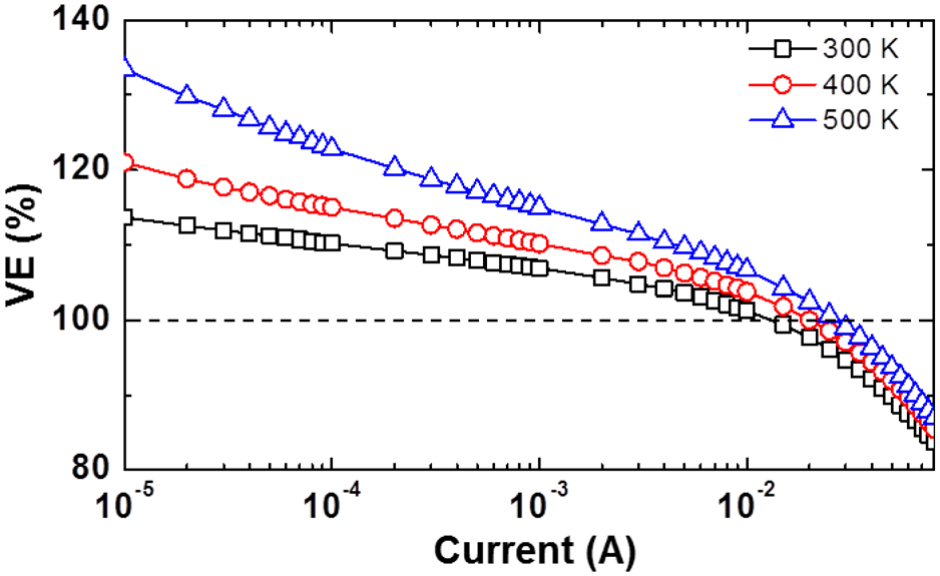
Voltage efficiency (VE) of sample under investigation as a function of driving current at various temperatures.

On the other aspect of efficiency of LEDs, we expect the VE > 100% when the carriers are energized more than the externally applied potential energy [recall *η*_VE_ = *hv*_*EL*_/*qV*]. As depicted in Fig. 10, the VE increases rapidly as an increase with elevated temperature in low current regime, which is a clear evidence proving that carriers in MQWs occupy higher energy state more than an applied potential energy. Notably, the maximum value of VE at 500 K seems to exceed the theoretical limitation^47^, which is derived based on the entropy analysis, indicating the unexpected large thermal energy transfer to the carriers. From the result in Fig. 10, the power efficiency (PE, *η*_PE_ and *η*_PE_ = *η*_EQE_ × *η*_VE_)^48^ in low current regime is expected to enhance even above 100% thanks to the additional energy supplying to carrier^13,43^.

From the experimental results and considerations discussed above, we can conclude as follows: (i) the SRH recombination increases with temperature, which well matches to theoretical prediction; (ii) the carriers in MQWs are energized by extrinsic factors, most likely by the SRH recombination and the lattice vibration, which is enhanced with elevated temperature; (iii) when the carriers gain sufficient energy to overcome the band offset from the extrinsic factors, the carrier escaping outside the MQWs is accelerated; (iv) the energized carriers induce the further reduction of radiative recombination probability; and (v) the reduction of IE and *B* accelerate the thermal droop.

On the basis of analysis and consideration, we revealed that all of three physical factors, i.e., *A*, *B*, and IE, simultaneously contribute to the thermal droop. From Ref.^49^, the carrier dynamics behind each physical factor can be estimated as followings. For *A*, the SRH recombination in MQWs, the carrier escaping from localized sites, and the carrier capture at barrier defect are possible mechanisms. In recent conventional blue LEDs, an alloy fluctuation during growth and a crystal quality of GaN barrier layer are well controlled, resulting in that the peak IQE reaches above 90%, as depicting in Fig. 2. Thus, we believe that the SRH recombination via defects in MQWs is a major cause due to the highest Indium mole fraction there. For the IE, the carrier escaping outside the MQWs via thermionic emission, the trap- and phonon-assisted tunneling, and the carrier emission to vacuum level by Auger recombination are possible mechanisms. Since the crystal quality of GaN barrier layer is well controlled, we estimated that the combination of Auger-assisted carrier emission and thermionic emission is a major cause to acceleration of the carrier escaping outside the MQWs. For *B*, in addition to the analysis given in this study, the QCSE due to strain and hole localization due to poor hole injection also could make an impact on the reduction of *B*. They also accelerate the carrier accumulation in energy space^3,28^, which consequently are in line with our analysis.

## Summary and conclusion

To elucidate the carrier dynamics behind the thermal droop in GaInN-based blue LEDs, we have looked deeper into the phenomenon by measuring DCLs as a function of driving current at various temperatures. Analysis of the values of *τ*_R_ and *τ*_NR_, obtained from the DCLs and the IQEs, have revealed the thermal droop is originated by simultaneous effects of the increase in SRH recombination, and the decrease in radiative recombination probability and the IE with elevated temperature. Notably, the temperature-dependent behavior of the SRH recombination well matches to theoretical prediction, while *B* is further reduced than theoretical prediction. Since the both are highly influenced by the distribution of carrier in real- and energy-space, we can infer that both are closely interrelated physical factors. To clarify the correlation between the two thermal droop mechanisms, we have conducted the thermodynamic analysis of the LED sample by investigating their *T*_C_ and *P*_HC_. Consequently, we have ascertained that the IE and the radiative recombination probability decreasing with temperature originate from the energized carriers in the MQWs. This indicates that the carriers in the MQWs energized by the SRH recombination and lattice vibration at high temperatures and currents accelerate the carrier escaping from the MQWs and the reduction of the radiative recombination probability. We believe that the analyses and considerations given in this study can provide further insights into the thermal droop in GaInN-based blue LEDs.

## Methods

### Sample preparation

For experiments, we prepared a GaInN-based blue LED grown by metalorganic vapor-phase epitaxy (Taiyo Nippon Sanso, EMC) on a *c*-plane flat sapphire substrate. The structure was conventional^46^, consisting of an LT-buffer layer, an undoped-GaN template layer, a Si-doped n-GaN layer, a 10-pair GaInN/GaN superlattice layer, a spacer layer, and 5 pairs of MQWs (*λ*_peak_ ≈ 450 nm at 300 K). On top of the MQWs, an electron-blocking AlGaN layer, a Mg-doped p-GaN layer, and a heavily Mg-doped p^+^-GaN contact layer were sequentially grown. The samples were fabricated to be chips with lateral electrodes (chip size = 300 μm × 300 μm) and packaged as surface-mount devices.

### Experimental measurement

The current–voltage (*I–V*) curves were measured using a Keithley 2602 sourcemeter. The light output power (LOP) and electroluminescence (EL) spectrum from LEDs were collected via a Si photodiode (Hamamatsu S2281) and a spectrometer (Avantes AvaSpec-2048), respectively. The DCLs were measured using an impedance analyzer (Agilent 4294A), where the four-terminal-fixture method was used for calibration to ensure accurate impedance values over a wide frequency range (40 Hz–110 MHz). The impedance data were measured via a voltage source mode with AC test signal of 20 mV (RMS) to ensure the reliability of experimental data. All measurements were conducted in a chamber to control and maintain the operating temperature under pulsed-current driving conditions (pulse period = 100 μs and duty cycle = 1%) to avoid self-heating.

## Data Availability

The datasets generated during and/or analyzed during the current study are available from the corresponding author on reasonable request.
